# Supplementary material for: Methods for improving the identification of acute stroke during ambulance calls: A scoping review
Source: PLoS One. 2025 Aug 13;20(8):e0327653. doi: 10.1371/journal.pone.0327653 (PMC12349133; doi:10.1371/journal.pone.0327653)
Supplement: S1 Appendix — (DOCX) [file pone.0327653.s001.docx]

**Appendix (1):**

# Embase

Embase Classic+Embase <1947 to 2024 Week 27>

| **#** | **Query** | **Results from 8 Jul 2024** |
| --- | --- | --- |
| 1 | Stroke.mp. | 602,372 |
| 2 | cerebrovascular accident/ or exp ischemic stroke/ | 340,636 |
| 3 | brain infarction/ | 68,096 |
| 4 | thrombotic stroke.mp. | 593 |
| 5 | cerebrovascular event*.mp. | 14,393 |
| 6 | brain ischemia/ or exp transient ischemic attack/ | 204,755 |
| 7 | hyperacute stroke*.mp. | 935 |
| 8 | acute Stroke*.mp. | 37,936 |
| 9 | cerebral stroke*.mp. | 3,180 |
| 10 | cerebral infarction*.mp. | 31,242 |
| 11 | cerebral ischemia.mp. | 46,059 |
| 12 | Large Vessel Occlusion*.mp. | 8,897 |
| 13 | Embolic Stroke*.mp. | 5,542 |
| 14 | (LVO or TIA or CVA or CVE).mp. | 38,785 |
| 15 | (Assess* or Diagnos* or Recogn* or Detect* or Identif* or Manag* or Screen* or Strati* or Evaluat* or Suspect*).mp. | 24,178,007 |
| 16 | ("emergency medical dispatch*" or "emergency medical service* communication* system*" or "ambulance control center*" or "ambulance control centre*" or "emergency communication center*" or "emergency communication centre*" or "Emergency Operations Centre*" or "Emergency Operations Center*").mp. | 1,193 |
| 17 | ("control room*" or "call handler*" or "call center*" or "call centre*" or "emergency call system*").mp. | 3,083 |
| 18 | ("Dispatch*" or "Dispatcher*" or "Dispatch Center*" or "Dispatch Centre*").mp. | 7,119 |
| 19 | ("medical priority dispatch system*" or "911" or 9-1-1 or Hotline*).mp. | 26,362 |
| 20 | 1 or 2 or 3 or 4 or 5 or 6 or 7 or 8 or 9 or 10 or 11 or 12 or 13 or 14 | 814,635 |
| 21 | 16 or 17 or 18 or 19 | 35,941 |
| 22 | 15 and 20 and 21 | 1,517 |

# Medline

Ovid MEDLINE(R) ALL <1946 to July 05, 2024>

| **#** | **Query** | **Results from 8 Jul 2024** |
| --- | --- | --- |
| 1 | exp stroke/ or exp brain infarction/ or exp ischemic stroke/ | 182,936 |
| 2 | exp embolic stroke/ | 453 |
| 3 | cerebrovascular accident*.mp. | 8,769 |
| 4 | cerebrovascular event*.mp. | 8,609 |
| 5 | exp brain ischemia/ or exp ischemic attack, transient/ | 128,127 |
| 6 | hyperacute stroke*.mp. | 422 |
| 7 | acute stroke*.mp. | 20,180 |
| 8 | cerebral stroke*.mp. | 1,967 |
| 9 | exp Cerebral Infarction/ | 37,533 |
| 10 | cerebral ischemia.mp. | 31,190 |
| 11 | Large Vessel Occlusion*.mp. | 4,784 |
| 12 | (LVO or TIA or CVA or CVE).mp. | 17,342 |
| 13 | (Assess* or Diagnos* or Recogn* or Detect* or Identif* or Manag* or Screen* or Strati* or Evaluat* or Suspect*).mp. | 17,353,879 |
| 14 | ("emergency medical dispatch*" or "emergency medical service* communication* system*" or "ambulance control center*" or "ambulance control centre*" or "emergency communication center*" or "emergency communication centre*" or "Emergency Operations Centre*" or "Emergency Operations Center*").mp. | 2,446 |
| 15 | ("control room*" or "call handler*" or "call center*" or "call centre*" or "emergency call system*").mp. | 1,782 |
| 16 | ("Dispatch*" or "Dispatcher*" or "Dispatch Center*" or "Dispatch Centre*").mp. | 4,849 |
| 17 | ("medical priority dispatch system*" or "911" or 9-1-1 or Hotline*).mp. | 19,127 |
| 18 | 1 or 2 or 3 or 4 or 5 or 6 or 7 or 8 or 9 or 10 or 11 or 12 | 271,569 |
| 19 | 14 or 15 or 16 or 17 | 26,527 |
| 20 | 13 and 18 and 19 | 501 |
| 21 | limit 20 to English language | 475 |

# Scopus

The search query ( TITLE-ABS-KEY ( stroke* OR "thrombotic stroke*" OR "hyperacute stroke*" OR "acute stroke*" OR "ischemic stroke*" OR "cerebral stroke*" OR "Embolic Stroke*" ) OR TITLE-ABS-KEY ( "brain infarction*" OR "brain ischemia" OR "cerebrovascular accident*" OR "cerebrovascular event*" OR "cerebral infarction*" OR "cerebral ischemia" ) OR TITLE-ABS-KEY ( "transient ischaemic attack*" OR "Large Vessel Occlusion*" ) OR TITLE-ABS-KEY ( "LVO" OR "TIA" OR "CVA" OR "CVE" . ) AND TITLE-ABS-KEY ( assess* OR diagnos* OR recogn* OR detect* OR identif* OR manag* OR screen* OR strati* OR evaluat* OR suspect* ) AND TITLE-ABS-KEY ( "emergency medical dispatch*" OR "emergency medical service* communication* system*" OR "ambulance control center*" OR "ambulance control centre*" OR "emergency communication center*" OR "emergency communication centre*" OR "Emergency Operations Centre*" OR "Emergency Operations Center*" ) OR TITLE-ABS-KEY ( "control room*" OR "call handler*" OR "call center*" OR "call centre*" OR "emergency call system*" ) OR TITLE-ABS-KEY ( "Dispatch*" OR "Dispatcher*" OR "Dispatch Center*" OR "Dispatch Centre*" ) OR TITLE-ABS-KEY ( "medical priority dispatch system*" OR "911" OR 9-1-1 OR hotline* ) ) from 1964 to 2024

Contains 668 document results.

# Web of Sciences

# Web of Science Search Strategy (v0.1)

# Database: Web of Science Core Collection

# Entitlements:

- WOS.IC: 1993 to 2024

- WOS.CCR: 1985 to 2024

- WOS.SCI: 1900 to 2024

- WOS.AHCI: 1975 to 2024

- WOS.BHCI: 2005 to 2024

- WOS.BSCI: 2005 to 2024

- WOS.ESCI: 2015 to 2024

- WOS.ISTP: 1990 to 2024

- WOS.SSCI: 1900 to 2024

- WOS.ISSHP: 1990 to 2024

# Searches:

Search: #1 AND #2 AND #7 Date Run: Tue Jul 02 2024 11:33:06 GMT+0100 (British Summer Time) Results: 812

Search: #6 OR #5 OR #4 OR #3 Date Run: Tue Jul 02 2024 11:32:57 GMT+0100 (British Summer Time) Results: 79499

Search: TS=("medical priority dispatch system*" or "911" or 9-1-1 or Hotlines) Date Run: Tue Jul 02 2024 11:32:02 GMT+0100 (British Summer Time) Results: 22162

Search: TS=("Dispatch*" or "Dispatcher*" or "Dispatch Center*" or "Dispatch Centre*") Date Run: Tue Jul 02 2024 11:31:47 GMT+0100 (British Summer Time) Results: 49364

Search: TS=("control room*" or "call handler*" or "call center*" or "call centre*" or "emergency call system*") Date Run: Tue Jul 02 2024 11:31:27 GMT+0100 (British Summer Time) Results: 8385

Search: TS=("emergency medical dispatch*" or "emergency medical service* communication* system*" or "ambulance control center*" or "ambulance control centre*" or "emergency communication center*" or "emergency communication centre*" or "Emergency Operations Centre*" or "Emergency Operations Center*") Date Run: Tue Jul 02 2024 11:31:11 GMT+0100 (British Summer Time) Results: 653

Search: TS=(Assess* OR Diagnos* OR Recogn* OR Detect* OR Identif* OR Manag* OR Screen* OR Strati* OR Evaluat* OR Suspect*.) Date Run: Tue Jul 02 2024 11:30:18 GMT+0100 (British Summer Time) Results: 25870578

Search: ((TS=(stroke* OR “thrombotic stroke*” OR "hyperacute stroke*" OR "acute stroke*" OR “ischemic stroke*" OR "cerebral stroke*" OR “Embolic Stroke*”)) OR TS=("brain infarction*" OR "brain ischemia" OR "cerebrovascular accident*" OR “cerebrovascular event*” OR "cerebral infarction*" OR “cerebral ischemia")) OR TS=(LVO OR TIA OR CVA OR CVE) Date Run: Tue Jul 02 2024 11:29:34 GMT+0100 (British Summer Time) Results: 551514

# CINAHL

| **#** | **Query** | **Limiters/Expanders** | **Last Run Via** | **Results** |
| --- | --- | --- | --- | --- |
| S5 | S1 AND S2 AND S3 | **Expanders** - Apply equivalent subjects  **Narrow by Language:**- english  **Search modes** - Find all my search terms | Interface - EBSCOhost Research Databases Search Screen - Advanced Search Database - CINAHL Plus | 254 |
| S4 | S1 AND S2 AND S3 | Expanders - Apply equivalent subjects Search modes - Find all my search terms | Interface - EBSCOhost Research Databases Search Screen - Advanced Search Database - CINAHL Plus | 260 |
| S3 | "( emergency medical dispatch OR emergency medical service communication system OR ambulance control center OR ambulance control centre OR emergency communication center OR emergency communication centre OR Emergency Operations Centre OR Emergency Operations Center ) OR ( control room OR call handler OR call center OR call centre OR emergency call system ) OR ( Dispatch OR Dispatcher OR Dispatch Center OR Dispatch Centre ) OR ( medical priority dispatch system OR 911 )" | Expanders - Apply equivalent subjects Search modes - SmartText Searching | Interface - EBSCOhost Research Databases Search Screen - Advanced Search Database - CINAHL Plus | 118,550 |
| S2 | "assess OR assessment OR assessing OR ( diagnosis or diagnosing or diagnoses ) OR ( recognition or recogni?e or recogni?ing ) OR ( detecting or detect or detection ) OR ( identify or identification or identifying ) OR ( manage or managing or management ) OR ( screen or screening ) OR ( stratification or stratify ) OR ( evaluation or evaluate or evaluating ) OR suspect" | Expanders - Apply equivalent subjects Search modes - SmartText Searching | Interface - EBSCOhost Research Databases Search Screen - Advanced Search Database - CINAHL Plus | 298,727 |
| S1 | "(stroke OR thrombotic stroke OR hyperacute stroke OR acute stroke OR ischemic stroke OR cerebral stroke OR Embolic Stroke) OR (brain infarction OR brain ischemia OR cerebrovascular accident* OR cerebrovascular event OR cerebral infarction OR cerebral ischemia) OR (transient ischaemic attack OR Large Vessel Occlusion) OR ( LVO OR TIA OR CVA OR CVE)" | Expanders - Apply equivalent subjects Search modes - SmartText Searching | Interface - EBSCOhost Research Databases Search Screen - Advanced Search Database - CINAHL Plus | 51,059 |
